# Effects of root spatial distribution on the elastic-plastic properties of soil-root blocks

**DOI:** 10.1038/s41598-017-00924-z

**Published:** 2017-04-11

**Authors:** Yunpeng Li, Yunqi Wang, Yujie Wang, Chao Ma

**Affiliations:** 1 School of Soil and Water Conservation at Beijing Forestry University, Beijing, 100083 China; 2 Soil and Water Conservation of Beijing Engineering Research Center at Beijing Forestry University, Beijing, 100083 China

## Abstract

Plant roots significantly influence soil properties, especially in soil beyond the limited area surrounding the main root stem. Some bias results may be generated if plastic properties of soil are merely used in evaluating slope stability without considering the effects of plant roots. In this research, effects of root spatial distribution on the elastic-plastic characteristics of soil-root blocks were examined. Triaxial tests and the Duncan-Chang model were used to analyze the correlation between root spatial characteristics and soil elastic-plastic properties. Safety factors of vegetated slopes were calculated to investigate the effect of roots on slope stability. The limit stress of remoulded soil was 103.52% to 231.61% greater than undisturbed soil in shallow soil layers. Increased root quantity led to an increased the failure ratio of soil bulk and the initial tangent modulus increased with root diameter. When calculating the safety factor of vegetated slopes, soil indexes for soil beyond the small cylinder surrounding the main stem should be properly considered to avoid safety factor overestimation.

## Introduction

Shallow landslides in the Three Gorge area of China have caused serious damage to landscapes^1, 2^ and have disrupted the traffic flow. Vegetation planting is a useful method for improving slope stability. The effects of plants on slope stabilization have attracted considerable research attentions^3^. Trees can influence slope stability through the weight of their aboveground parts and the anchoring ability of roots^4, 5^. Cohesion enhancement, water pressure decreases, and root reinforcement forces are major topics in analyzing of vegetation effects on slope stability^6, 7^. In evaluating plant root effects on slope stability, an additional cohesion force due to roots occurs in a cylindrical area around the main stem (CATM) of trees. Consideration of this force can provide more reliable safety evaluations. Under field conditions typically, there are many roots in the area beyond the CATM. Soil beyond this limited area is commonly regarded as a plastic material that can have unpredictable effects on slope stability. Effects of roots on the soils in these areas are usually ignored or de-emphasized, which can also lead to inaccurate stability evaluation^8^.

The number and diameter-class distribution of roots is closely related to soil depth, plant species, and environmental heterogeneity^9^. Approaches such as experimental investigation^10, 11^, theoretical derivation^8, 12, 13^ and numerical simulation^14–16^ have been used in analyzing of roots on soil shear strength. Shear tests and Mohr-Coulomb strength theory are commonly applied to explore interactions between roots and soil^11, 17^. These tests provide useful results in the CATM. However, root growth is usually irregular in the area beyond the CATM, leading to an unclear evaluation of root growth characteristics^18, 19^. Shear tests include the influence of root angles on soil shear strength measurements^20^ and cannot be used to describe the effects of roots on soil in the area beyond the CATM. Triaxial tests provide a more realistic measure of strength, because they measure root effects without considering the root angles. Triaxial tests consider soil-root blocks as elastic-plastic rather than plastic, and can provide a good estimate of the effects of roots on soil shear strength.

In this work, triaxial tests and the DuncanChang model were used to analyze the elastic-plastic properties of soil-root blocks. Root characteristics (number and diameter) were investigated within defined soil depths of selected vegetation stands. Triaxial tests on undisturbed and remoulded soil-root blocks were conducted in the laboratory. Vegetated slopes of different sizes were built to calculate the safety factors. This research aims to: (1) analyze root spatial distribution at different soil depths in different plant stands, (2) determine the effects of plant roots on soil elastic-plastic properties, (3) study the differences in stability of soil-root blocks within five stands, (4) compare the safety factors of vegetated slope related to root spatial distribution characteristics.

## Results

### Characteristics of the root spatial distribution in different stands

**Figure 1:**
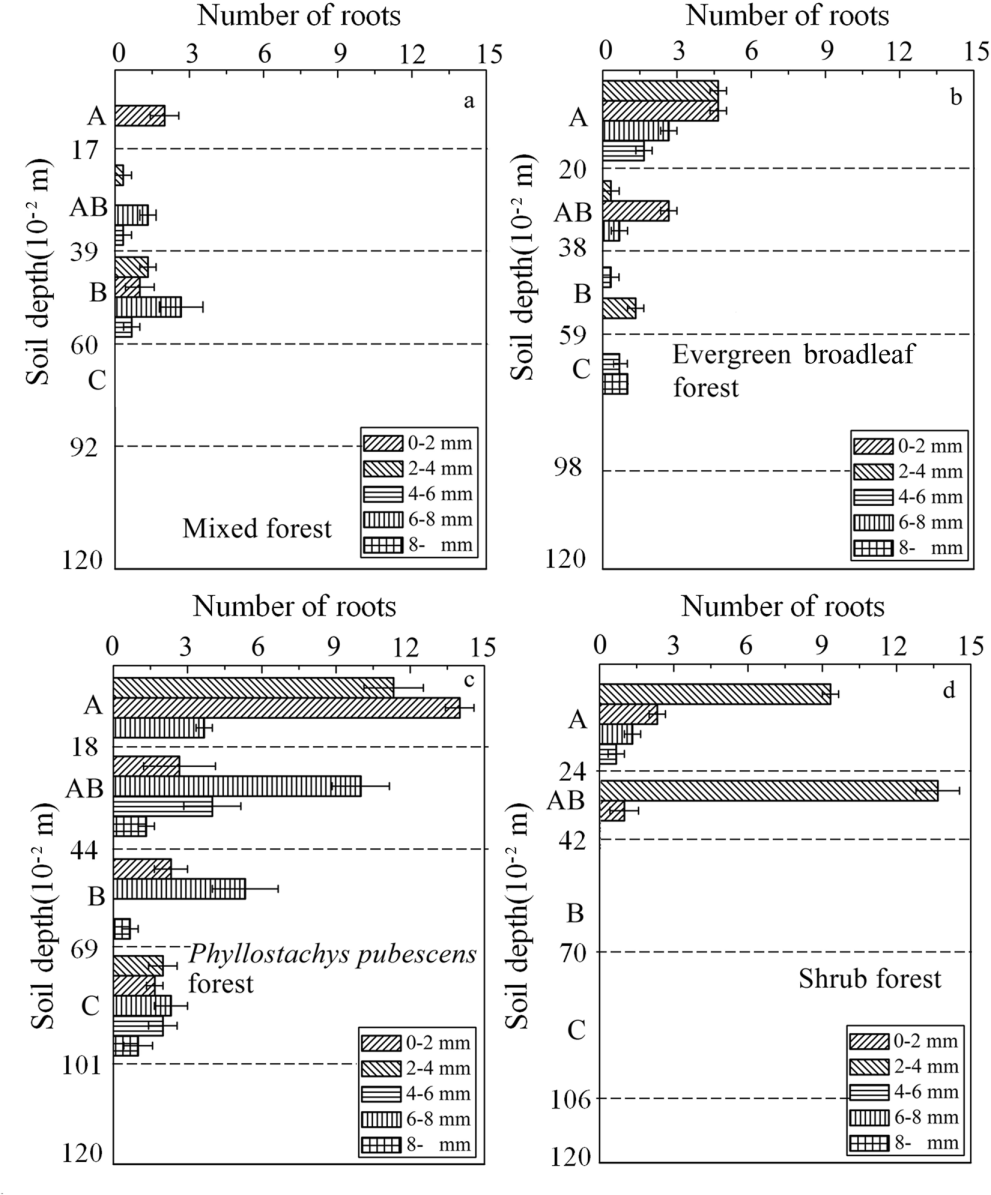
Comparisons between number of roots and root diameter-class of soil layers from 4 stands. Error bars indicate the standard deviation (SD) of each histogram.

Number and diameter-class of roots within four stands are shown in Fig. 1. *Phyllostachys pubescens* forest has the largest numbers of root, followed by shrub forest, and evergreen broadleaf forest. Roots are only found in soil layer C in *Phyllostachys pubescens* forest (Fig. 1c). There are no roots in soil layers B and C in the shrub forest. Number and diameter-class of roots increase with soil depth in mixed forest while number and diameter-class of roots decrease with soil depth in the evergreen broadleaf forest and shrub forest. Mixed forest, *Phyllostachys pubescens* forest and evergreen broadleaf forest have a higher proportion of root with diameters >2 mm (Fig. 1a and b). Root diameters <2 mm usually exist in the *Phyllostachys pubescens* forest and shrub forest.

### Soil shear strength of different stands

**Figure 2:**
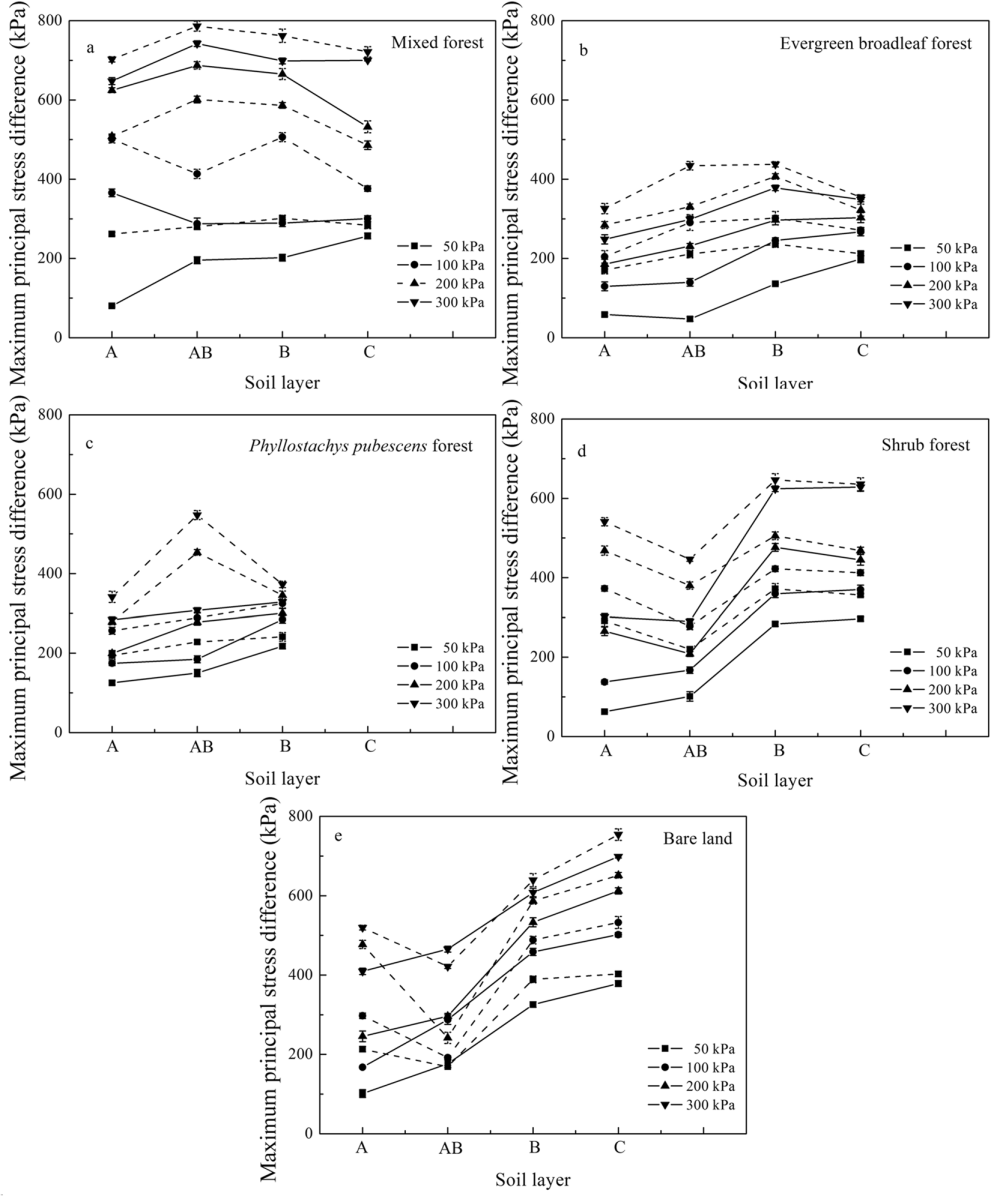
Variation of limit stress in soil layers under different confining pressures using the triaxial test. The solid line depicts the UU test on undisturbed soil and the dotted line depicts the UU test on remoulded soil. Error bars indicate the standard deviation (SD) of each point.

Soil shear strength depends a great extent on the principal stress, which can be used to represent the strength of elastoplastic soil blocks. When soil failure occurs, the maximum principal stress can be deemed as the limit stress that is different in the soil layers of stands (Fig. 2, Supplementary File 1). Maximum limit stress of 800 kPa was approximately found in soil layer AB of the mixed forest (Fig. 2a). Limit stresses of evergreen broadleaf forest are generally small (peaks did not exceed 420 kPa). Limit stresses in *Phyllostachys pubescens* forest and bare land increase with soil depth under arbitrary confining pressure. When the confining pressure approaches 300 kPa, there is an obvious drop on limit stress in soil layer C of the mixed forest and evergreen broadleaf forest, and in soil layer B of the shrub forest (confining pressure was 200 kPa) (Fig. 2d). However, the limit stress significantly increases from soil layer AB to B under different confining pressures except in the mixed forest.

Compared with undisturbed soil limit stress in shallow soil layers (layers A and AB), the limit stress of remoulded soil significantly increase. Limit stress of remoulded soil is 103.52% to 231.61% greater than undisturbed soil. Remoulded soil in shrub forest has the largest increasement of mean limit stress (70%) (Fig. 2d, Supplymentary file 1). However, in soil layers B and C, no obvious differences were found between undisturbed and remoulded soil limit stress. Tree roots are probably the main cause of the differences between the limit stress of undisturbed soil and remoulded soil in both soil layers A and AB. Limit stress of undisturbed and remoulded soil tend to be similar with reduction of soil root quantity. No difference exists between the limit stresses of undisturbed and remoulded soil in bare land except for soil layer A (Fig. 2e).

### Elastic-plastic model of different stands

The *c* and *ϕ* were obtained by the consolidated undrained triaxial test (CU) using remoulded soil (Table 1,﻿ Supplemen﻿tary File 1). The *c* is maximum in soil layers B and C of each stand except for *Phyllostachys pubescens* forest and bare land. The *c* in evergreen broadleaf forest increases linearly with soil depth but decreases with soil depth in *Phyllostachys pubescens* forest. The *ϕ* randomly varies among the five stands, and its value ranged from 20° to 28°. *R*_*f*_ indicates the failure ratio of soil blocks. When failure occurred, those with 1 < *R*_*f*_ could have remaining strength. *R*_*f*_ in soil layers AB and B mostly exceed 1. In other soil layers, the value of *R*_*f*_ is all less than 1. *E*_*0*_ varies with soil depth. *E*_*0*_ in the mixed forest are greater than the other stands. Although much of root mass exist in the shallow soil layer, there are no significant differences in *E*_*0*_ between bare land and the other stands.

**Table 1:** Elastic-plastic parameters of five stand soil.

Stands	Soil layer	*c* (kPa)	*ϕ* (°)	(*σ*_*1*_ − *σ*_*3*_)_*f*_ (kPa)	*R*_*f*_	*E*_*0*_ (MPa)	*R* ^*2*^
Mixed forest	A	55.6	24.4	242.9	0.71	3.21	0.86
	AB	83.2	25.1	335.4	0.98	18.00	0.87
	B	128.4	26.1	490.3	1.00	34.67	0.74
	C	96.7	27.1	399.9	0.92	18.99	0.87
Evergreen broadleaf forest	A	35.2	27.9	204.9	1.06	3.37	0.80
	AB	45.6	22.3	197.1	1.10	2.16	0.79
	B	58.1	24.2	249.1	1.20	7.41	0.84
	C	78.2	26.2	330.3	1.11	15.76	0.83
*Phyllostachys pubescens* forest	A	57.2	23.6	241.6	1.08	3.06	0.81
	AB	35.6	26.2	193.4	0.92	3.06	0.83
	B	26.1	22.8	141.8	0.90	2.49	0.84
	C	—	—	—	—	—	—
Shrub forest	A	17.2	27.5	142.5	0.99	1.79	0.78
	AB	62.2	18.6	219.9	1.20	3.10	0.81
	B	118.7	24.1	435.3	1.10	16.49	0.87
	C	16.0	26.9	134.7	0.95	2.02	0.85
Bare land	A	59.6	20.5	225.7	1.04	3.81	0.80
	AB	42.0	25.4	207.9	1.13	3.12	0.83
	B	20.0	27.1	149.1	1.00	1.42	0.78
	C	55.7	21.0	217.9	0.96	3.65	0.87

**Figure 3:**
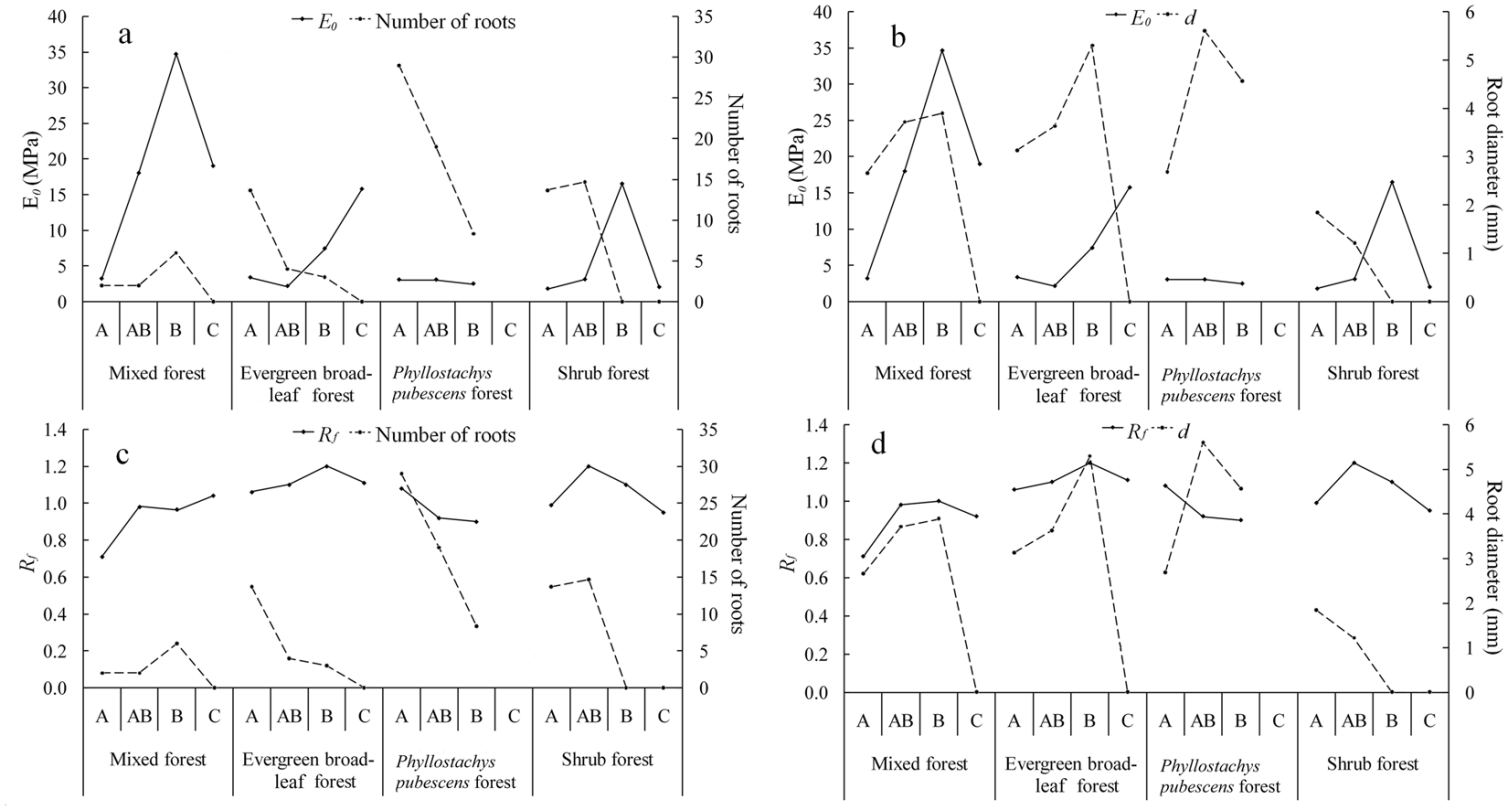
Correlations between root spatial characteristics (mean diameter and number of roots) and soil elastic-plastic properties (*R*_*f*_ and *E*_*0*_).

Roots in soil-root blocks have limitation effects on soil elastic-plastic behavior (Fig. 3 and Table 2). *E*_*0*_ decrease as root quantity increase where not in mixed forest and *Phyllostachys pubescens* forest (Fig. 3a). Similar trends can be found between root quantity and *R*_*f*_. Root diameter is positively correlated with *E*_*0*_, but no obvious correlation exists between root diameter and *R*_*f*_. In addition, soil properties also have impact on elastic-plastic properties. *E*_*0*_ and *R*_*f*_ increase as soil bulk density increase but decrease as soil moisture content increase. In general, elastic strength of soil-root blocks weakens as root quantity increase (Fig. 3b and d). However, the presence of roots with large diameters might enhance the elastic strength of soils.

**Table 2:** Correlation of soil elastic-plastic properties with root properties and soil properties.

Parameters	Stands	Correlation coefficient (*r*)			
		Number of roots	Root diameter	Soil bulk density	Soil moisture content
*E*_*0*_	Mixed forest	0.87	1^**^	1^**^	−1^**^
	Evergreen broadleaf forest	−0.50	0.50	0.50	−0.50
	*Phyllostachys pubescens* forest	0.87	0	−0.87	−0.87
	Shrub forest	1^**^	−1^**^	1^**^	−1^**^
*R*_*f*_	Mixed forest	0.87	1^**^	1^**^	−1^**^
	Evergreen broadleaf forest	−1^**^	1^**^	1^**^	−1^**^
	*Phyllostachys pubescens* forest	1	−0.50	−1^**^	−1^**^
	Shrub forest	1	−1^**^	1^**^	−1^**^

Spearman correlation analysis is used in this paper.

**Represents significance level <0.01; *Represents significance level <0.05.

### Comparisons of safety factors between two conditions

**Figure 4:**
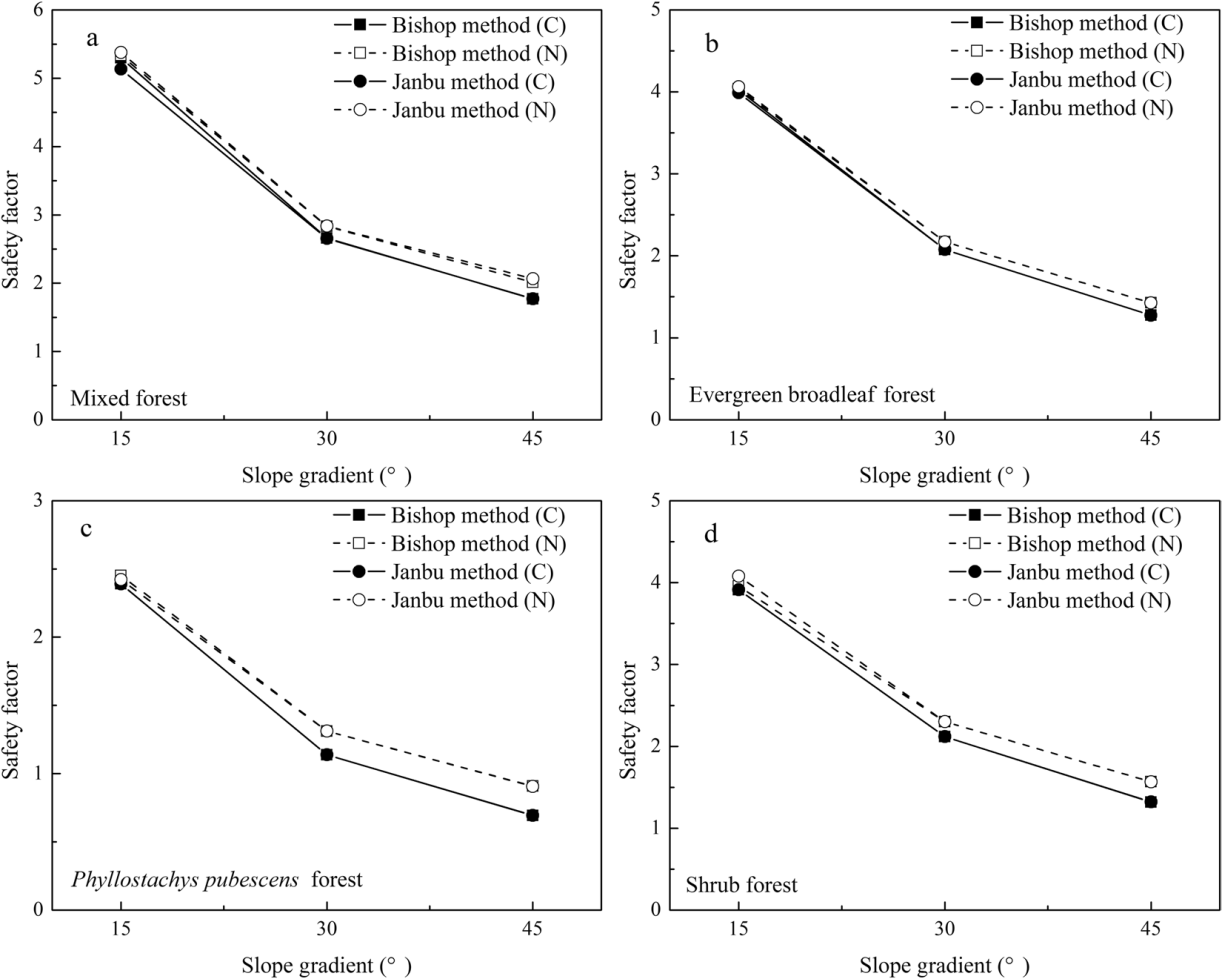
Safety factors in different slope gradients of 4 plant stands. C Indicates calculations that include root effects; N Indicates that root effects were not considered in calculations.

Influence of different soil properties on slope stability calculations are shown in Fig. 4. Soil properties (***γ***, *c*, *ϕ*, *c*′ and *ϕ*′) used in the calculation are the average value of each soil layer within one stand. Safety factors of the four stands all exceed 1.1 except *Phyllostachys pubescens* forest at 45°. Safety factors of vegetated slopes gradually decrease as slope gradient. However, safety factors considering the effects of roots are smaller than those without root effects. Increasing safety factor percentages for different slope gradients (15°, 30° and 45°) are 0.42% to 4.71%, 4.08% to 15.20% and 7.98% to 31.02% when root effects were not considered, respectively. The largest increase in safety factor is 31.02% in *Phyllostachys pubescens* forest (Fig. 4c). Root effects have great impact on slope stability calculations, and roots tended to reduce the safety factors. This effect increases with slope gradient. When root effects are not considered, the largest safety factor is 5.38 in mixed forest (Fig. 4a), followed by 4.06 in evergreen broadleaf forest (Fig. 4b), and 4.02 in shrub forest (Fig. 4d). Considering a stable slope of *F* = 1, the smallest safety slope gradient is 33° when root effects are considered, but 37° when root effects are not considered.

## Discussion

### Composition of root spatial distributions in the different stands

Number and diameter of roots of each stand were recorded. Soil profile layers were used to ensure the same soil properties within each soil layer^21^. Only *Phyllostachys pubescens* forest roots extended to soil layer C, which was mainly due to the growth habits of *Phyllostachys pubescens* ^22^. Many roots exist in soil beyond the CATM. Roots of *Phyllostachys pubescens* had grown in relation to each other and increased with soil depth. Similar findings were reported by Gale and Grigal^23^. Soil layer A of the mixed forest only has 2–4 mm diameter roots. *Pinus massoniana* and *Cinnamomum camphora* are the dominant tree species in this study area^24^. The roots of these species spread away from the main stem but do not extend beyond the CATM. Small trees and low shrubs exist in the evergreen broadleaf forest and shrub forest. McMichael and Quisenberry^25^ demonstrated that small trees and low shrubs had strong interspecific competition, with roots extending over a wide area to obtain water and nutrients. Therefore, a wider root diameter distribution (0 to 8 mm) was found between small trees and low shrubs. Root diameter distribution reduces with increasing of soil depth due to limited root growth of trees in the evergreen broadleaf forest and the shrub forest. The mixed forest with large trees shows a variable root diameter distribution. Roots of shrub forest plants appeared only in soil layers A and AB, indicating moderate interactions between the plant roots. At deeper soil depths (50–100 cm), roots of the evergreen broadleaf forest and the mixed forest connect and intertwine, resulting in a greater root diameter class distribution in soil beyond the CATM.

### Limit stress of the different stands

The limit stresses in soil layers within stands increase with confining pressure in the UU test on undisturbed and remoulded soil samples. *In situ* limit stresses of bare land increase with soil depth and confining pressure. Regardless of stands, soil depths and confining pressures (were 50 kPa, 100 kPa, 200 kPa and 300 kPa), limit stresses of remoulded soil samples were all higher than those of undisturbed soil. The main distinctions of undisturbed soil and remoulded soil samples are the original soil structure and friction between soil and root, which was shown in previous research^26–28^. Giadrossich *et al*.^29^ found that friction between soil and roots was an important factor enhancing soil stability. We found that the loosening effect of roots on soil structure^30, 31^ decreased the shear strength of undisturbed soil samples compared to that of remoulded soil samples. Friction between soil and roots do not substantially contribute to failure resistance. Compared to the other stands, limit stresses of bare land in soil layer AB are unchanged in undisturbed and remoulded soil samples. The bare land was once farmland^32^ and agricultural activities compacted the soil^33^, leading to a greater limit stress behavior in soil layer AB.

Shrub forests have a larger root quantity in shallow soil layer. Limit stresses of undisturbed and remoulded soil samples reversed in soil layers A and AB (the greatest average increase rate of 70%). Root growth is highly developed in soil layers A and AB. However, shear resistance was less in undisturbed soil samples. Many studies^34–37^ found, based on direct shear tests, that roots reinforce soil shear strength. However, in a natural environment, radial forces are not properly applied to roots. This means that roots might not be fully functional in the shear process. In this study, roots were randomly distributed in the soil samples of the triaxial tests. These tests indicated a decrease in the limit stress of undisturbed soil samples though the soil contained many roots. For soil layer B, roots have little effect on loosening the soil, which result in similar limit stress values of undisturbed and remoulded soil samples. Decrease of limit stress in soil layer C may result from the evaluation method of limit stress (when the peak appeared, we scored axial stress when the axial strain reached 30% of the maximum strain). Variation of the limit stresses of undisturbed and remoulded soil samples indicates that the effects of roots on soil loosening result in a decrease in shear resistance. Therefore, safety factors might be overestimated if the effects of roots on soil beyond the CATM are not considered.

### Variation of soil strength in different stands

*R*_*f*_ and *E*_*0*_ were derived from the Duncan-Chang model. *R*_*f*_ is a measure of the failure ratio of soil-root blocks. When *R*_*f*_ < 1, the soil-root blocks retain some strength based on the value of *R*_*f*_ after shear failure. For smaller values of *R*_*f*_, the retained strength of soil-root blocks is larger. When *R*_*f*_ > 1, the strength of soil-root blocks is zero after shear failure. The larger the *R*_*f*_ value is, the more susceptible the soil-root blocks breaks. Variations of elastic-plastic characteristics of soil-root blocks within stands were also compared by *E*_*0*_. *E*_*0*_ measures the strength of deformation resistance. The higher the *E*_*0*_ value is, the greater the degree of rigidity becomes. As applied to soil-root blocks, the *E*_*0*_ is the ability to resist deformation. Roots possess strong flexibility and the combination of roots and soil improves soil elastic-plastic properties. This was also reported by Franck *et al*.^38^. In pull out and shear tests, large diameter roots provided strong resistance to shear failure. Similar conclusions were obtained in other papers^39, 40^. Excessive root content makes soil-root blocks more susceptible to plastic deformation and less amenable to original structure restoration. Many studies have concluded that roots increase soil shear strength^35, 36, 41–43^. In the soil beyond the CATM, influences of roots on soil shear strength are difficult to evaluate owing to random distribution of the roots. In this study, we consistently found that roots weakened the elastic-plastic performance of soil-root blocks due to their loosening effect. Therefore, the stability of soil-root blocks was reduced by the presence of roots.

For vegetated slopes, safety factor estimates considering the effects of roots are smaller than those that do not consider roots using either the Janbu^44^ or Bishop^45^ methods. Safety factor estimate differences range from 0.42% to 31.05%. Cohesive force and soil friction angle are the main factors influencing the safety factor estimates. In this study, the effect of roots on soil is converted to the change of cohesive force and friction angle, which were also reported in Li *et al*.^16^ and Ji *et al*.’s^46^ studies. Based on the results of Fig. 2 and Table 1, roots have great impact the performance of soil shear strength beyond the CATM. Roots weakened the cohesive force but enhance the soil friction angle due to their elastic strength. When the slope gradient is small, the differences in slope stability were not significant when the effects of roots are either considered or ignored. However, greater differences exist when the slope gradient is steep. If a slope reaches the critical value of the safety factor, evaluations on this slope without considering root effects might result in a conclusion inconsistent with the actual safety of the situation. Calculations without considering the impact of roots can produce upwardly biased safety factor.

## Conclusion

Using the undisturbed and remoulded soil samples from different soil layers, influences of plant roots on the elastic-plastic properties of soil-root blocks were analyzed. Root spatial distributions within plant stands were compared. Safety factors of vegetated slopes were calculated using two methods to investigate effects of roots on slope stability. Roots decreased the elastic-plastic strength of soil-root blocks. The greater the root quantity is, the more susceptible plastic deformation the soil-root blocks performs. Soil deformation resistance increased when larger diameter roots were present in the soil. In calculations of the safety factor of vegetated slopes, safety factors without consideration of root effects were greater than those where root effects were considered. Thus, indexes of soil beyond the CATM should be carefully considered to avoid overestimation of the safety factor.

## Materials and Methods

### Study site

**Figure 5:**
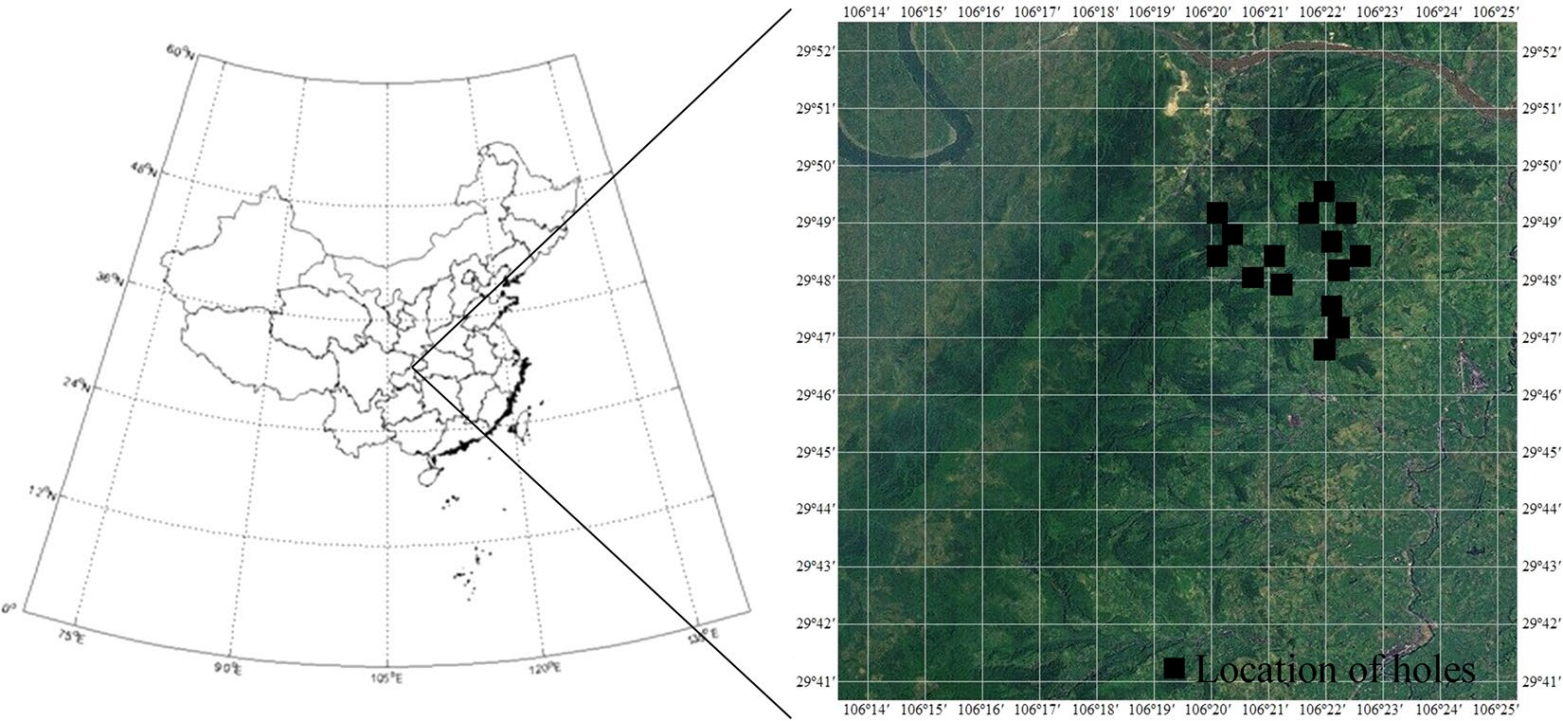
Location of Jinyun Mountain in Chongqing, China. (Left: ArcGIS, Version 9.3. http://www.esri.com/; Right: Google Earth, Version 7.1. http://google-earth.en.softonic.com/. Figure 5 was made by Yunpeng Li).

The study area is located in Jinyun Mountain in Chongqing Beibei (Fig. 5). It has a subtropical monsoon humid climate with mean annual temperature of 13.6 °C. The highest elevation is 951 m. Due to the high mean annual rainfall (1783.8 mm), there is a large area of evergreen broadleaf forest. Four stands (mixed forest, evergreen broadleaf forest, *Phyllostachys pubescens* forest, and shrub forest) were selected as the plots. Bare land has been chosen as the control group. Soil in Jinyun Mountain area is derived from Triassic Xujiahe Formation sandstone and shale. Soil types are Orthic Acrisols and a small amount of Aric Anthrosols^47^. All of the holes were evenly distributed on the slopes of Jinyun Mountain with an average degree of 5°.

### Collection of soil samples

In each stand, 3 holes were dug to 80–120 cm soil depth. Soil samples were collected at A (surface soil layer), AB (transition layer), B (subsoil layer) and C (substratum) layers in the 5 stands (including the control stand). Each hole provided 4 undisturbed using a soil sampler with a 61.8-mm inside diameter and 125 mm long. Soil samples were wrapped with saran wrap to minimize loss of moisture. Disturbed soil with a weight of approximately 2 kg for each soil layer was collected in ziplock bags. A total of 15 holes were dug and 60 soil samples of undisturbed soil and 120 kg of disturbed soil were collected, respectively. Other soil parameters for each soil layer of the five stands are listed in Table 3.

**Table 3:** Parameters of soil properties.

Stands	Soil layer	Mixed forest	Evergreen broadleaf forest	*Phyllostachys pubescens* forest	Shrub forest	Bare land
Soil bulk density *γ* (g cm^−3^)	A	1.15	1.18	1.14	0.73	0.99
	AB	1.39	1.49	1.46	1.17	1.12
	B	1.51	1.69	1.54	1.46	1.2
	C	1.72	1.53	1.64	—	1.46
Soil moisture content *w* (%)	A	19.3	17.46	18.82	13.92	19.45
	AB	18.04	14.94	17.45	16.23	18.18
	B	16.32	14.25	14.5	17.91	16.93
	C	14.65	13.96	13.78	—	11.75

### Plant composition and root characteristics

Common species of four typical forest stands are shown in Table 4. *Pinus massoniana* and *Gordonia acuminate* in the mixed forest are mature trees with heights ranging from 8 to15 m. Low shrubs and small trees predominated in the shrub forest. A high density of *Lindera kwangtunensis* and *Cunninghamia lanceolata* were evenly distributed in this stand.

**Table 4:** Major components of four plant stands.

Stands	Species
Mixed forest	*Gordonia acuminata*, *Symplocos setchuensis*, *Adinandra bockiana*, *Symplocos lancifolia*, *Neolitsea aurata*, *Diospyros morrisiana*, *Woodwardia japonica*, *Lophatherum gracile*.
Evergreen broadleaf forest	*Gordonia acuminata*, *Neolitsea aurata*, *Castanopsis carlesii*, *Indigofera esquirolii*
*Phyllostachys pubescens* forest	*Phyllostachys pubescens*, *Maesa japonica*, *Sarcandra glabra*, *Oplismenus compositus*, *Commelina communis*
Shrub forest	*kwangtunensis Lindera*, *Eurya japonica*, *Alniphyllum fortune*, *Machilus pingii*, *Cunninghamia lanceolata*, *Hemercocallis fulva*, *Conyza canadensis*

**Figure 6:**
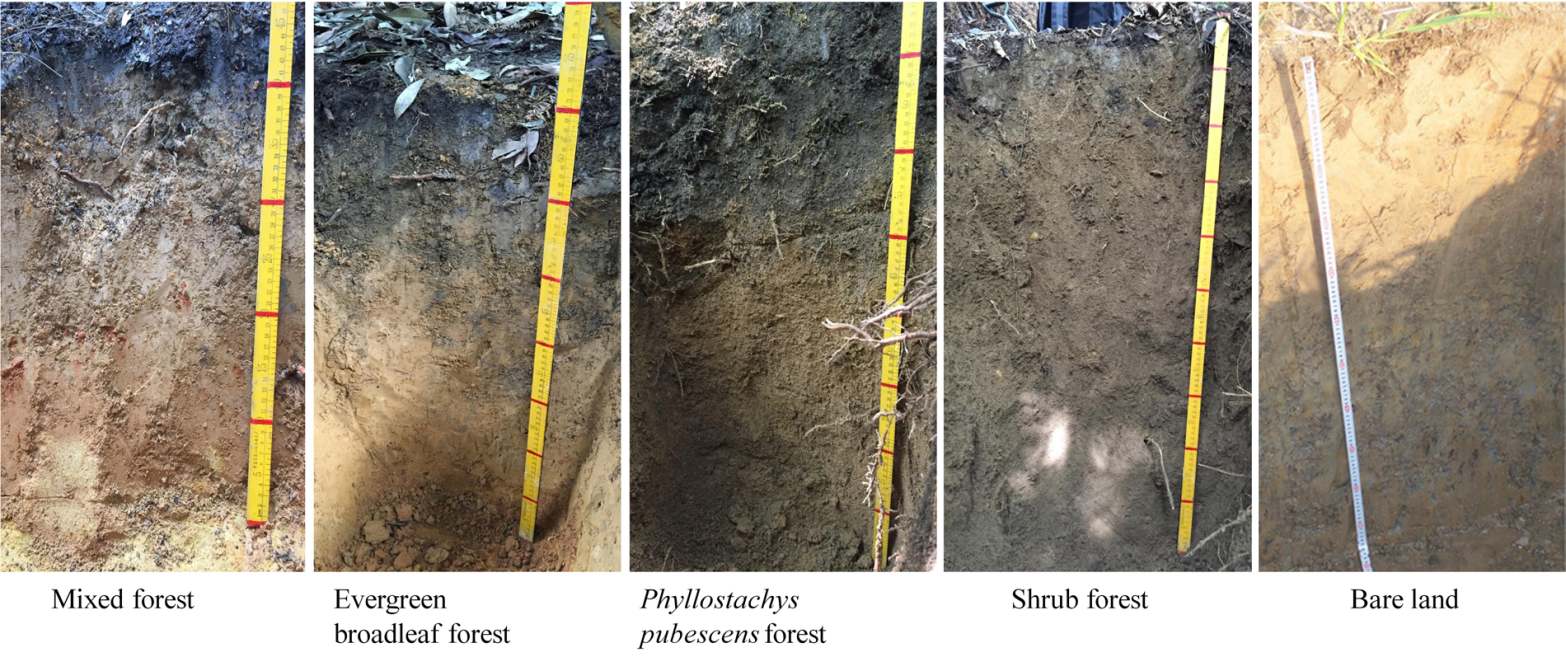
Soil profiles of different stands.

Root diameter and root quantity of each soil sample were measured. Roots exposed in the soil profile were removed and counted. Root diameters were divided into 5 groups: 0–2 mm, 2–4 mm, 4–6 mm, 6–8 mm and above 8 mm. Root quantity in each group was counted. Soil profiles of different stands in field are shown in Fig. 6.

### Calculations of soil shear strength

In order to give a more realistic-approaching strength of soil-root blocks, we introduce triaxial test to analysis elastic-plastic properties of distributed and remoulded soil samples. Total soil shearing resistance for unsaturated soils is calculated as

1 $$S=c^{\prime} +({\mu }_{a}-{\mu }_{w})\tan \,{\varphi }^{b}+(\sigma -{\mu }_{a})\tan \,\varphi ^{\prime} +{\rm{\Delta }}S,$$

where *S* = total soil shearing resistance (kPa), *c*′ = effective cohesion (kPa), *μ*_*a*_ = pore-air pressure (kPa), *μ*_*w*_ = pore-water pressure (kPa), *ϕ* ^*b*^ = angle describing the increase in shear strength due to an increase in matric suction (*μ*_*a*_ *−* *μ*_*w*_) (◦), *σ* = normal stress on the shear plane (kPa), *ϕ*′ = effective soil friction angle (◦), and ∆*S* = increase in shear strength due to roots (kPa). In the process of shearing in field, a small number of macropores caused by plant and animal activities weakened *S* in soil. The real soil shearing resistance would be less than the value calculated by Eq. (1). In order to get close to the actual situation and for computing convenience, we modified Eq. (1) as

2 $$S={S}_{s}+{S}_{w}+{\rm{\Delta }}S-{S}_{p},$$

where *S*_*s*_ = shearing resistance of pure soil (kPa), *S*_*w*_ = shearing resistance caused by pore-water pressure (kPa), *S*_*p*_ = decrease shearing resistance caused by soil pores (kPa). In order to obtain values of the above parameters, a triaxial shear test (TSZ30-2.0 Shanghai Research Institute of Materials, China) was applied in the experiment. Shear rate was 0.09 mm/min. Confining pressures (*σ*_*3*_) were 50 kPa, 100 kPa, 200 kPa and 300 kPa. The shearing process continued to 5% of the axial strain after the peak occurred. If a peak was not appear, we defined the axial stress was peak when axial strain reached 30% of the maximum strain. *S* was measured by the unconsolidated undrained triaxial test (UU) on undisturbed soil samples. For disturbed soil, latex tubing was used to make remoulded soil samples. After cutting into the required shape (61.8 mm diameter and 125 mm long), each soil sample was consolidated under confining pressure of 50 kPa by a triaxial shear tester. Then, the consolidated undrained triaxial test (CU) and UU were applied on the remoulded soil samples to obtain the soil shear strength index of *c* and *ϕ* and the soil shearing resistance (*S*_*s*_ + *S*_*w*_ + ∆*S*), respectively.

### Elastic-plastic model

The shear failure of soil-root blocks is a continuous process. In order to investigate the nonlinear deformation of soil, we used the Duncan-Chang model^48^ to investigate the shear failure process of soil-root blocks beyond the CATM. This model is a variable-modular elastic model built on incremental generalized version of Hooke’s law. The Duncan-Chang model can reflect the nonlinear deformation of soil and also express soil elastic-plastic deformation. There were no stones with diameters >2 mm in the study area soils providing suitable conditions for the Duncan-Chang model. In addition, roots were assumed to be evenly distributed in the soil.

The relationship between (*σ*_*1*_ − *σ*_*3*_) and *ε*_*1*_, based on Kondner’s hyperbola^49^ was

3 $$({\sigma }_{1}-{\sigma }_{3})={\varepsilon }_{1}/(a+b{\varepsilon }_{1}),$$

where (*σ*_*1*_ − *σ*_*3*_) = deviatoric compressive pressure (kPa), *ε*_*1*_ = axial strain, *a*, *b* = constant. When soil failure occurred ((*σ*_*1*_ − *σ*_*3*_)_*f*_), failure ratio *R*_*f*_ was expressed as

4 $${R}_{f}={({\sigma }_{1}-{\sigma }_{3})}_{f}/{({\sigma }_{1}-{\sigma }_{3})}_{ult},$$

where (*σ*_*1*_ − *σ*_*3*_)_*ult*_ = ultimate deviatoric compressive pressure (kPa). Then the Mohr-Coulomb Failure Criterion was used:

5 $${({\sigma }_{1}-{\sigma }_{3})}_{f}=(2c\,\cos \,\varphi +2{\sigma }_{3}\,\sin \,\varphi )/(1-\,\sin \,\varphi ).$$

when *ε*_*1*_ → ∞, (*σ*_*1*_ − *σ*_*3*_) = (*σ*_*1*_ − *σ*_*3*_)_*ult*_ = 1/*b*, the initial tangent modulus *E*_*0*_^44^ was used in Eq. (3). The process of soil shear failure can be regarded as elastic failure. So Eq. (3) can be expressed as

6 $$({\sigma }_{1}-{\sigma }_{3})={\varepsilon }_{1}/(1/{E}_{0}+{\varepsilon }_{1}/{({\sigma }_{1}-{\sigma }_{3})}_{ult}).$$

If the peak failed to appear, we defined that the axial stress was (*σ*_*1*_ − *σ*_*3*_)_*f*_ when the *ε*_*1*_ value reached 30% of it. Then, *R*_*f*_ and *E*_*0*_ were calculated and analyzed as given below.

### Stability calculations of vegetated slope

**Figure 7:**
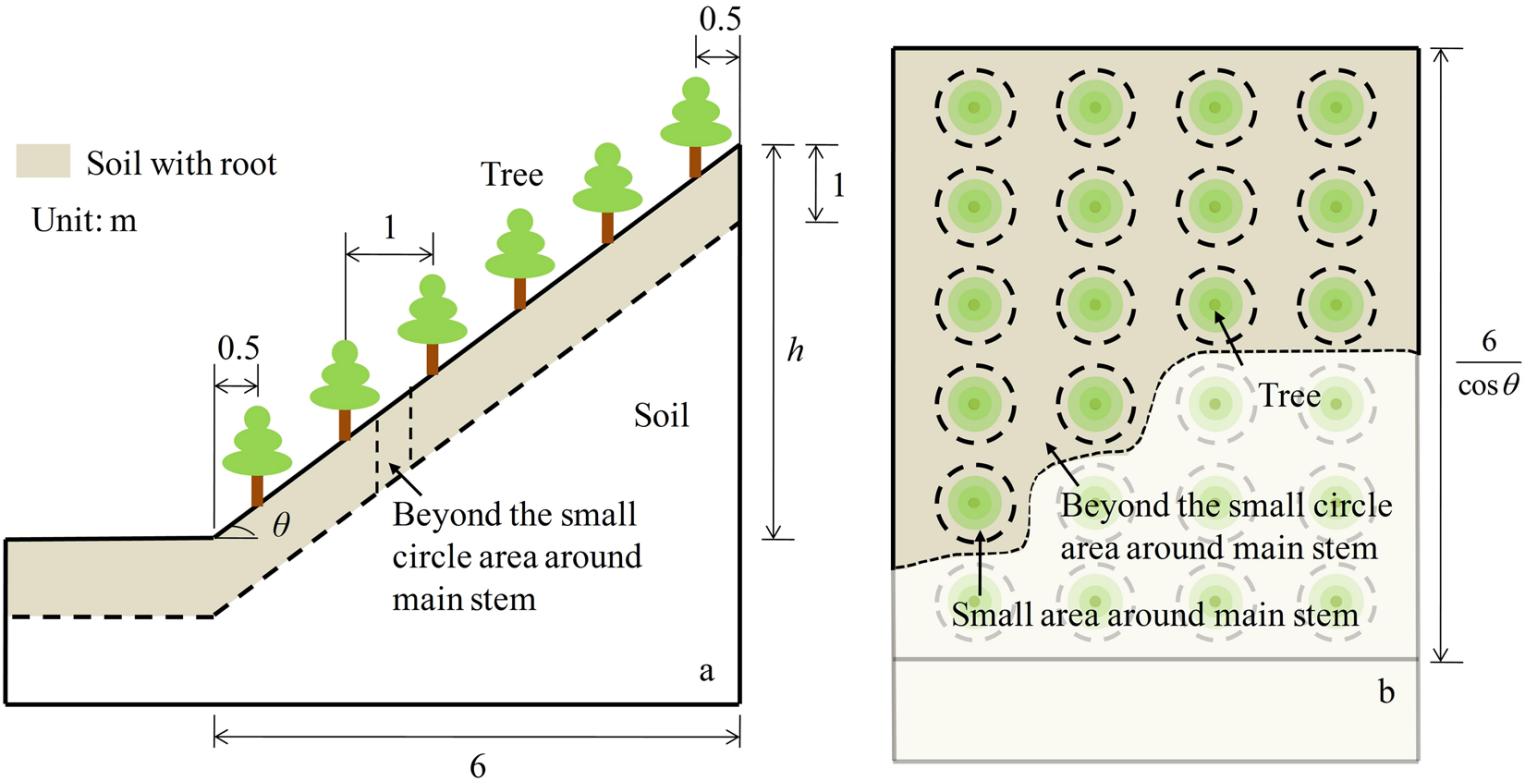
Geometric profile of vegetated slopes. Lateral (**a**) and aerial views (**b**) of slope are illustrated.

To explore the influence of plant roots on slope stability, vegetated slopes were built to calculate the safety factors (*F*) (Fig. 7a). The homogenous soil-slopes were composed of trees and soil. Soil properties in Table 1 were used in calculation. For the trees on slopes, the point load model was simulated as a weight of 20 KN in the vertical direction. Six trees were planted on slope separated by horizontal distance of 1 m. Slope gradients (*θ*) were 15°, 30° and 45°. The horizontal length of the slope was 6 m ensuring that the horizontal distance between trees was constant. Cohesive force and friction angle tested by the triaxial tests could be divided into two groups: total *c* and *ϕ*, effective cohesive force *c*′ and soil friction angle *ϕ*′. The total *c* and *ϕ* represented the soil indexes of the soil-root blocks. The *c*′ and *ϕ*′ were soil indexes without roots. We assumed that the influence of roots on soil shear strength beyond the CATM extended to 1 m (Fig. 7b). All the calculations were completed using a program from Lizheng Software (Tianjin, China). Methods of Janbu^44^ and Bishop^45^ were used to calculate the slope safety factor.

## Electronic supplementary material

Supplementary files
