# Supplementary material for: Effects of root spatial distribution on the elastic-plastic properties of soil-root blocks
Source: Sci Rep. 2017 Apr 11;7:800. doi: 10.1038/s41598-017-00924-z (PMC5429779; doi:10.1038/s41598-017-00924-z)
Supplement: Supplementary file 1 — Supplementary files [file 41598_2017_924_MOESM1_ESM.pdf]

Effects of root spatial distribution on the elastic-plastic properties of soil-root blocks

Yunpeng Li[1], Yunqi Wang[1, 2], Yujie Wang[1, 2], Chao Ma[1]

1. *School of Soil and Water Conservation at Beijing Forestry University, Beijing, 100083, China.*2. *Soil and Water Conservation of Beijing Engineering Research Center at Beijing Forestry University，Beijing 100083, China*

Correspondence to: Yunqi Wang (wangyunqibj@163.com)

Title page:

Yunpeng Li

School of Soil and Water Conservation at Beijing Forestry University, Beijing, 100083, China

303156042@qq.com

Yunqi Wang

Soil and Water Conservation of Beijing Engineering Research Centerat Beijing Forestry University，Beijing 100083, China, School of Soil and Water Conservation at Beijing Forestry University, Beijing, 100083, China

wangyunqibj@163.com

Yujie Wang

Soil and Water Conservation of Beijing Engineering Research Centerat Beijing Forestry University，Beijing 100083, China, School of Soil and Water Conservation at Beijing Forestry University, Beijing, 100083, China

wangyujiebj@126.com

Chao Ma

Soil and Water Conservation of Beijing Engineering Research Centerat Beijing Forestry University，Beijing 100083, China, School of Soil and Water Conservation at Beijing Forestry University, Beijing, 100083, China

sanguoxumei@163.com

Corresponding author

Yunqi Wang

School of Soil and Water Conservation at Beijing Forestry University, Beijing, 100083, China
Postal address: Beijing Forestry University, Tsinghua east road no. 35. Haidian district, 100083, Beijing, China.

wangyunqibj@163.com

Supplementary for Fig. 1

	Soil layer	Diameter class (mm)	R-1 Number of roots	R-2 Number of roots	R-3 Number of roots	Average
Mixed forest	A	0–2	0	0	0	0
		2–4	1	2	3	2
		4–6	0	0	0	0
		6–8	0	0	0	0
		>8	0	0	0	0
	AB	0–2	1	0	0	0.333333
		2–4	0	0	0	0
		4–6	1	2	1	1.333333
		6–8	0	0	1	0.333333
		>8	0	0	0	0
	B	0–2	1	1	2	1.333333
		2–4	2	1	0	1
		4–6	1	3	4	2.666667
		6–8	1	0	1	0.666667
		>8	0	0	0	0
	C	0–2	0	0	0	0
		2–4	0	0	0	0
		4–6	0	0	0	0
		6–8	0	0	0	0
		>8	0	0	0	0

	Soil layer	Diameter class (mm)	R-1 Number of roots	R-2 Number of roots	R-3 Number of roots	Average
Evergreen broadleaf forest	A	0-2	5	5	4	4.666667
		2-4	4	5	5	4.666667
		4-6	3	3	2	2.666667
		6-8	1	2	2	1.666667
		>8	0	0	0	0
	AB	0-2	0	0	1	0.333333
		2-4	3	3	2	2.666667
		4-6	1	1	0	0.666667
		6-8	0	0	0	0
		>8	1	0	0	0.333333
	B	0-2	2	2	0	1.333333
		2-4	0	0	0	0
		4-6	0	0	0	0
		6-8	0	1	1	0.666667
		>8	1	1	1	1
	C	0-2	0	0	0	0
		2-4	0	0	0	0
		4-6	0	0	0	0
		6-8	0	0	0	0
		>8	0	0	0	0

	Soil layer	Diameter class (mm)	R-1 Number of roots	R-2 Number of roots	R-3 Number of roots	Average
Phyllostachys pubescens forest	A	0–2	9	13	12	11.33333
		2–4	15	14	13	14
		4–6	4	4	3	3.666667
		6–8	0	0	0	0
		>8	0	0	0	0
	AB	0–2	0	0	0	0
		2–4	5	3	0	2.666667
		4–6	8	10	12	10
		6–8	4	6	2	4
		>8	2	1	1	1.333333
	B	0–2	0	0	0	0
		2–4	1	3	3	2.333333
		4–6	8	4	4	5.333333
		6–8	0	0	0	0
		>8	1	1	0	0.666667
	C	0–2	1	2	3	2
		2–4	2	2	1	1.666667
		4–6	3	3	1	2.333333
		6–8	2	1	3	2
		>8	0	1	2	1

	Soil layer	Diameter class (mm)	R-1 Number of roots	R-2 Number of roots	R-3 Number of roots	Average
Shrub forest	A	0−2	9	9	10	9.333333
		2−4	2	3	2	2.333333
		4−6	1	1	2	1.333333
		6−8	1	1	0	0.666667
		>8	0	0	0	0
	AB	0−2	15	14	12	13.66667
		2−4	0	2	1	1
		4−6	0	0	0	0
		6−8	0	0	0	0
		>8	0	0	0	0
	B	0−2	0	0	0	0
		2−4	0	0	0	0
		4−6	0	0	0	0
		6−8	0	0	0	0
		>8	0	0	0	0
	C	0−2	0	0	0	0
		2−4	0	0	0	0
		4−6	0	0	0	0
		6−8	0	0	0	0
		>8	0	0	0	0

Supplementary for Fig. 2

Evergreen broadleaf forest								
R-1	UU (undisturbed)	UU (undisturbed)	UU (undisturbed)	UU (undisturbed)	UU（remoulded）	UU（remoulded）	UU（remoulded）	UU（remoulded）
A	59.92	144.05	203.24	259.99	184.92	225.94	280.19	344.35
AB	45.54	126.01	235.77	302.4	224.86	316.31	339.84	449.6
B	133.02	247.64	313.41	378.8	235.07	325.96	402.95	437.8
C	186.65	253.61	286.33	346.58	220.34	280.98	301.14	355
R-2	UU (undisturbed)	UU (undisturbed)	UU (undisturbed)	UU (undisturbed)	UU（remoulded）	UU（remoulded）	UU（remoulded）	UU（remoulded）
A	62.32	128.49	176.85	252.16	162.58	188.86	296.41	316.65
AB	54.61	146.74	236.54	306.42	206.14	267.89	328.46	426.5
B	138.42	235.62	289.3	386.42	246.13	289.64	401.25	445.8
C	200.16	270.12	306.41	343.21	208.16	268.42	332.16	362.1
R-3	UU (undisturbed)	UU (undisturbed)	UU (undisturbed)	UU (undisturbed)	UU（remoulded）	UU（remoulded）	UU（remoulded）	UU（remoulded）
A	52.6	116.5	176.8	232.6	165.2	198.4	276.6	316.7
AB	41.6	146.2	222.1	286.8	202.6	287.6	323.2	426.5
B	136.5	252.7	287.9	369.5	226.5	289.5	416.5	429.5
C	208.6	278.2	316.2	356.4	208.4	265.4	333.3	346.7

Mixed forest								
R-1	UU (undisturbed)	UU (undisturbed)	UU (undisturbed)	UU (undisturbed)	UU（remoulded）	UU（remoulded）	UU（remoulded）	UU（remoulded）
A	80.85	351.94	499.71	635.14	264.3	502.8	631.4	699.2
AB	182.98	300.67	605.84	737.89	276.9	429.6	683.4	793.6
B	193.64	300.26	591.02	706.71	295.2	489.9	648.2	745.4
C	261.22	290.31	481.22	701.44	280.5	374.6	535.8	703.4
R-2	UU (undisturbed)	UU (undisturbed)	UU (undisturbed)	UU (undisturbed)	UU（remoulded）	UU（remoulded）	UU（remoulded）	UU（remoulded）
A	76.8	372.3	512.6	652.1	265.2	512.2	625.4	712.2
AB	202.2	295.2	608.5	752.3	285.4	408.3	700.2	796.5
B	213.5	289.2	592.1	700.2	312.6	512.5	682.4	785.2
C	262.8	308.5	500.4	698.2	296.4	385.4	548.3	729.3
R-3	UU (undisturbed)	UU (undisturbed)	UU (undisturbed)	UU (undisturbed)	UU（remoulded）	UU（remoulded）	UU（remoulded）	UU（remoulded）
A	83.1	372.5	512.2	655.5	255.6	488.6	616.4	696.4
AB	201.2	267.2	589.5	736.2	278.3	402.6	678.6	768.5
B	198.2	278.5	576.2	688.8	296.1	516.2	665	756.3
C	246.5	302.6	475.3	701.2	275.4	368.6	512.2	732.1

Phyllostachys pubescens forest								
R-1	UU (undisturbed)	UU (undisturbed)	UU (undisturbed)	UU (undisturbed)	UU（remoulded）	UU（remoulded）	UU（remoulded）	UU（remoulded）
A	121.43	172.96	207.82	293.69	184	244.3	281.2	322.1
AB	148.99	191.35	276.83	305.61	221.3	269.3	452.8	531.5
B	222.43	279.64	295.61	331.58	225.8	308.3	335.5	373.7
C								
R-2	UU (undisturbed)	UU (undisturbed)	UU (undisturbed)	UU (undisturbed)	UU（remoulded）	UU（remoulded）	UU（remoulded）	UU（remoulded）
A	132.3	182.4	201.2	278.5	208.2	265.5	285.3	355.3
AB	162.1	190.2	288.8	316.5	231.3	301.3	462.3	555.5
B	222.2	297.6	316.2	333.2	251.6	332.3	360.2	382.6
C								
R-3	UU (undisturbed)	UU (undisturbed)	UU (undisturbed)	UU (undisturbed)	UU（remoulded）	UU（remoulded）	UU（remoulded）	UU（remoulded）
A	122.2	168.2	189.6	280.5	190.4	260.3	269.3	346.5
AB	139.6	172.4	269.63	300.3	232.3	296.4	444.5	555.5
B	208.2	276.8	289.6	320.6	246.5	333.2	340.2	362.1
C								

Shrub forest								
R-1	UU (undisturbed)	UU (undisturbed)	UU (undisturbed)	UU (undisturbed)	UU（remoulded）	UU（remoulded）	UU（remoulded）	UU（remoulded）
A	62.34	129.64	280.38	292.46	272.2	372.9	477.2	536.2
AB	84.77	158.43	209.53	284.51	220.1	266.2	371.1	445.8
B	283.75	367.71	483.95	629.89	383.8	418.6	491.5	659.7
C	297.31	377.17	425.82	640.37	355	405.9	456.5	658
R-2	UU (undisturbed)	UU (undisturbed)	UU (undisturbed)	UU (undisturbed)	UU（remoulded）	UU（remoulded）	UU（remoulded）	UU（remoulded）
A	65.2	140.6	260.5	312.6	300.2	380.6	475.6	555.2
AB	106.32	178.2	216.5	300.2	226.4	282.3	392.5	452.3
B	290.4	365.2	482.6	628.2	378.6	432.1	512.6	655.4
C	302.6	378.6	456.2	629.3	362.3	422.5	472.8	628.6
R-3	UU (undisturbed)	UU (undisturbed)	UU (undisturbed)	UU (undisturbed)	UU（remoulded）	UU（remoulded）	UU（remoulded）	UU（remoulded）
A	60.2	142.5	255.5	299.5	302.1	364.3	452.1	531.6
AB	112.3	165.6	200.1	285.2	213.5	285.5	376.4	440.5
B	276.8	345.5	462.5	614.3	352.4	416.2	512.4	623.5
C	289.5	354.2	452.2	615.2	351.6	408.5	475.6	620.2

Bare land								
R-1	UU (undisturbed)	UU (undisturbed)	UU (undisturbed)	UU (undisturbed)	UU（remoulded）	UU（remoulded）	UU（remoulded）	UU（remoulded）
A	87.58	164.55	226.65	420.12	205.8	287.8	490.1	511.3
AB	190.34	270.45	300.61	475.43	170.8	189	221.8	428
B	333.81	467.13	549.01	623.08	399.3	487.5	587.5	662.1
C	383.55	497.4	623.23	693.73	397.5	548.9	659.6	774.8
R-2	UU (undisturbed)	UU (undisturbed)	UU (undisturbed)	UU (undisturbed)	UU（remoulded）	UU（remoulded）	UU（remoulded）	UU（remoulded）
A	106.5	168.2	255.1	408.3	211.2	302.6	476	523.6
AB	168.5	296.3	286.4	460.2	165.3	188.5	252.3	420.5
B	316.52	446.2	526.3	601.2	378.2	476.7	596.3	623.3
C	368.2	498.3	608.2	702.2	398.4	512.1	642.5	746.5
R-3	UU (undisturbed)	UU (undisturbed)	UU (undisturbed)	UU (undisturbed)	UU（remoulded）	UU（remoulded）	UU（remoulded）	UU（remoulded）
A	110.3	170.2	255.2	400.2	222.3	302.1	465.2	524.2
AB	170.6	296.2	302.4	460.3	170.6	198.5	251.3	416.3
B	326.8	462.5	523.6	600.2	390.4	500.4	576.3	632.5
C	384.2	509.58	605.2	700.2	412.5	536.5	652.1	741.6

Supplementary for Fig. 4

	Soil layer	Soil bulk density (g cm^3)	soil moisture content (%)	c (kPa)（CU）	ϕ (°) (CU)	c' (kPa)（CU）	ϕ' (°) (CU)	slope gradient (°)	Heigh (m)
Mixed forest	A	1.15	19.30	55.60	24.40	30.20	32.00	15	1.607695
	AB	1.39	18.04	83.20	25.10	79.40	26.70	30	3.464102
	B	1.51	16.32	128.40	26.10	122.80	27.50	45	6
	C	1.72	14.65	96.70	27.10	87.60	28.40		
Evergreen broadleaf forest	A	1.18	17.46	35.20	27.90	25.40	33.10	15	1.607695
	AB	1.49	14.94	45.60	22.30	25.70	31.20	30	3.464102
	B	1.69	14.25	58.10	24.20	53.10	26.90	45	6
	C	1.53	13.96	78.20	26.20	67.70	29.00		
Phyllostachys pubescens forest	A	1.14	18.82	57.20	23.60	26.70	35.00	15	1.607695
	AB	1.46	17.45	35.60	26.20	13.50	34.50	30	3.464102
	B	1.54	14.50	26.10	22.80	4.80	35.80	45	6
Shrub forest	A	0.73	13.92	17.20	27.50	15.30	42.00	15	1.607695
	AB	1.17	16.23	62.20	18.60	56.40	23.40	30	3.464102
	B	1.46	17.91	118.70	24.10	111.50	25.70	45	6
	C			16.00	26.90	14.10	35.60		
Bare land	A	0.99	19.45	59.60	20.50	55.80	24.40	15	1.607695
	AB	1.12	18.18	42.00	25.40	18.20	34.20	30	3.464102
	B	1.20	16.93	20.00	27.10	6.10	36.00	45	6
	C	1.46	11.75	55.70	21.00	38.20	30.10		
